# Quality of Life After Breast Cancer Surgery With or Without Reconstruction

**Published:** 2009-06-02

**Authors:** Demetris Stavrou, Oren Weissman, Anna Polyniki, Neofytos Papageorgiou, Joseph Haik, Nimrod Farber, Eyal Winkler

**Affiliations:** ^a^ The Department of Plastic & Reconstructive Surgery, The Chaim Sheba Medical Center, Sackler School of Medicine, Tel-Aviv University, Tel-Aviv, Israel; ^b^ Mental Health Services, Nicosia, Cyprus; ^c^ Department of Internal Medicine B, Sheba Medical Center, Tel-Hashomer, Tel-Aviv, Israel

## Abstract

In the modern era, where breast-conserving surgery is a viable alternative to mastectomy, breast cancer patients and their healthcare providers have to consider the issue of quality of life in regards to the type of surgery. The choice of surgical procedure should consider the perceptions of women diagnosed with breast cancer as well as their functional and emotional well-being. A more holistic approach to the patient should be implemented with proper psychological evaluation before and psychological support after the crisis.

Breast cancer is a major health issue in modern society. The National Cancer Institute estimates that 12.7% of women born today will be diagnosed with breast cancer during the course of their lifetime.^1^ Breast cancer can impact patients psychologically as well as organically, which can manifest as postmastectomy depression, increased anxiety, shame, and occasional ideas of suicide.^2^ Nowadays, breast-conserving treatments such as lumpectomy followed by radiation,^3^,^4^ or breast reconstruction after mastectomy,^5^ are viable alternatives to mastectomy alone, especially in early stages of the disease. Until the role of women in society and views on sexuality dramatically changed in the 60s and 70s of the 20th century, breast reconstruction was considered merely vain^6^ and performed in a troubled subset of cancer patients.^7^ Fortunately, this view has changed in recent decades, resulting in an ever-growing increase in patients choosing breast-conserving surgery or breast reconstruction. This trend has spawned efforts to assess the efficacy of these treatment modalities, taking into account not only mortality rates and reconstructive techniques but patients' satisfaction rates and quality of life (QOL) as well. As Donabedian argued more than 30 years ago, the ultimate valuator for quality of care is its effectiveness in achieving or producing health and satisfaction.^8^ In other words, care cannot be of high quality unless the patient is satisfied. In today's increasingly competitive healthcare marketplace, the issue of measuring the quality of care given to patients, as well as the QOL they derive from it, has become a topic of considerable interest and controversy among healthcare providers as well as patients. In this work, we attempt to highlight the perceptions of breast cancer survivors in regards to changes in their QOL after being diagnosed with breast cancer, as well as following their medical treatment, including surgery, chemo-radiotherapy, or both. The concept of body image, which is closely related to health-related QOL, will also be discussed.

## METHODS

The following databases were searched: PubMed, Cochrane Library, and Ovid by using the terms “quality of life” or “health related quality of life” or “breast cancer” or “breast reconstruction” or “body image.” The search was limited to publications with abstracts in the last 20 years and in English. In addition, citations within obtained papers were scrutinized to identify additional studies.

## RESULTS AND DISCUSSION

### Quality of Life

QOL is a term that is extensively used by sociologists, philosophers, economists, politicians, and healthcare providers. The term originates from Aristotle's *Nicomachean Ethics*, dating back to 330 bc, in which he recognizes the relation between QOL, happiness, and the subjective values of the individual (Aristotle, 335–323 bc). There is a wide and multidimensional definition for QOL, which relies heavily on patients' sex, age, ethnicity, and religious beliefs. It encompasses personal tastes, hobbies, experiences, perceptions, attitudes, and beliefs, all of which can be divided into 4 primary categories, physical and occupational, psychosocial, social, and somatic.^9^

Upon examination, there are several relevant variables in the application of the QOL measurement in healthcare (Health Related Quality of Life [HRQOL]). HRQOL measured by QALY (Quality Adjusted Life Years) refers to an individual's physical, functional, and emotional well-being, as well as fulfillment and satisfaction in aspects of life related to health. It is a “value assigned to the duration of life and modified by impairments,”^10^ considering the individual's own sense of well-being and not the healthcare provider's one.^11^

When quantifying the effects of medical and surgical treatments on patients' lives, healthcare providers use a variety of QOL assessment tools, usually in the form of questionnaires. Reliable and valid patient questionnaires are essential for aesthetic and reconstructive breast surgeons. Reliability, validity, and responsiveness are prerequisites for an ideal patient-reported outcome questionnaire, as outlined by the Scientific Advisory Committee of the Medical Outcomes Trust.^12^ A review of the literature by Pusic et al^12^ has identified 227 health-outcome questionnaires used in previous breast surgery studies. Only 1 QOL assessment tool, the Breast-Related Symptoms Questionnaire, demonstrated adequate development and validation in its target population, despite having significant limitations.^13^,^14^

Several studies have suggested that body image and feelings of attractiveness are improved following breast reconstruction,^15^–^17^ although mood state, uncertainty, distress, and overall QOL do not differ significantly.^18^–^20^ When comparing both treatment modalities discussed here, a meta-analysis of the relevant literature indicates a small advantage for breast-conserving surgery.^21^ Recent studies question the psychosocial effects of breast reconstruction and suggest that its outcome is not uniformly benign or beneficial. After examining 190 women with breast cancer, Yurek et al^22^ reported that patients treated with reconstructive surgery had lower rates of sexual activity and fewer signs of sexual responsiveness than those who had mastectomy or lumpectomy alone. An even broader study, surveying 1957 breast cancer surviving patients, suggested more positive QOL-related outcomes with women who underwent lumpectomy rather than mastectomy with or without reconstruction.^19^ When researchers compared HRQOL measurements, no differences were seen among both surgical groups, including the variable that evaluated patients' fear of recurrence. However, the reconstruction group had a less than expected benefit on body image and they were more likely to feel that breast cancer had a negative impact on their sex lives. The fear of recurrence mentioned above is a parameter that affects all patients, irrespective of the treatment type. This parameter seems to affect QOL assessment more than the choice of surgery.^23^–^25^

A prospective study that compared 3 breast cancer–associated surgical procedures (lumpectomy, mastectomy alone, and mastectomy with subsequent breast reconstruction), assessed QOL of patients 1, 3, 6, 12, 18, and 24 months after initial diagnosis was established.^26^ The results showed that patients who underwent lumpectomy or mastectomy with reconstruction had no better QOL than those who had mastectomy alone. A possible explanation for this finding might be that the stage of the disease was the driving force behind the decision to undergo mastectomy alone, precluding these patients from considering breast-conserving surgery. On the contrary, women who underwent breast reconstruction experienced greater mood disturbances as well as poorer feelings of well-being following surgery, an effect that persisted for 18 months after surgery. The postoperative pain, length of surgery and the length of hospitalization, and the absence from everyday activities tend to be greater with breast reconstruction. The fact that these factors impart a detrimental effect on QOL, especially in the short term, may be responsible for those findings.^26^

The majority of studies agree that among all the surgical groups, at least 1 year after the surgery, the psychosocial or health-related QOL are less determined by the primary surgery and more influenced by other factors such as age, exposure to adjuvant therapy, and other health problems.^27^,^28^ A possible yet considerable interference with these results can be the fact that breast reconstruction is more often done by younger, educated white women, married or in a relationship,^19^ suggesting the possibility of unequal access to healthcare.^16^

A recent study about the QOL in disease-free survivors of breast cancer, 5–10 years after their initial diagnosis, reported high levels of functioning and QOL, with minimal differences reflecting expected age-related changes. However, systemic adjuvant chemotherapeutic treatment was associated with poorer functioning on several aspects of QOL.^29^ Studies that focus on the impact of chemotherapy on women show a significant disruption of the physical,^30^,^31^ psychosocial,^32^,^33^ and sexual aspects of QOL.^33^,^34^ The fact that younger women are more likely to be candidates for aggressive chemotherapy with a detrimental impact on their QOL must also be considered.^35^ In addition to that, although it has been previously suggested that women who had mastectomy may be influenced more by chemotherapy,^30^ most of the studies do not consider the possible interactions between chemotherapy and surgery. Moreover, the premature onset of menopausal symptoms and ovarian failure propagated by the chemotherapy can cause disruption in the sexual lives of younger women,^36^ thus confounding results. The persistence of QOL disruption over time,^37^,^36^ along with the finding that social support decreases over time,^38^ indicates the need for long-term patient support beyond the initial phase of diagnosis and surgery.

Several studies have tried to compare different kinds of breast-reconstruction techniques regarding their effects on QOL. In a group of 63 patients who underwent breast reconstruction, 36 with pedicled flaps and 27 with free transverse rectus abdominis musculocutaneous flaps (TRAM), no difference was reported between the 2 subgroups with regards to HRQOL.^39^ Another study compared the effects on QOL in women with breast cancer seeking immediate breast reconstruction versus delayed breast reconstruction after mastectomy.^40^ It was reported that patients who elected immediate reconstruction suffer more severe disturbances in mental health status, emotional well-being, and higher levels of anxiety than women who underwent delayed reconstruction. These findings are in controversy with studies that support immediate reconstruction because of reduced costs, improved cosmesis,^5^ and less psychological disturbance than is otherwise observed in the early stages after mastectomy.^21^,^41^ The benefits seen in the delayed group can be explained by the growing evidence that shows emotional resiliency of postmastectomy women within the first year after surgery.^42^,^43^ These findings support the observation of greater satisfaction from surgical outcomes with patients who had longer periods of time between mastectomy and sequential breast reconstruction.^7^

When interpreting results from QOL studies with breast cancer patients, it is important to remember that information given to patients preoperatively about their options for surgery is not always optimal.^44^–^46^ Surgeon's preference, limitation in operating room time, and healthcare costs may bias preoperative counseling^47^ and consequently the patient's decision for the type of operation.

### Body Image

Another concept of major interest in breast cancer patients is body image. Body image has received many interpretations since it was first described in the 1920s, but the most familiar one is the definition by Price,^48^ who describes body image as “the totality of how one feels and thinks about one's own body and appearance.” She incorporates for the first time the 3 elements of body image: (1) *body reality*—“the body as it really exists”; (2) *body ideal*—“subjective picture of each person on how the body should look and perform”; (3) *body presentation*—“how the body is presented to the outside environment.” In our society's restrictive definition of *physical beauty*^49^ where beautiful people are considered more intelligent, outgoing, happier, and better company,^50^ breast surgery can greatly affect the body image and thus the QOL of a woman.

The breast is unique for women as it is associated with reproduction and nurturing as well as sexual appeal. Women with breast cancer have to deal not only with the trauma of disfigurement but also with the fear of rejection from their partners and loss of femininity. Breast cancer treatment has been suggested to change body reality and body ideal and may affect body presentation.^48^,^51^ Negative perceptions of body image among breast cancer survivors include dissatisfaction with appearance and surgical scars, reluctance to see one's naked body, and feelings of diminished sexual attractiveness.^52^–^54^

The literature suggests that women who underwent breast-conserving surgery demonstrate a more positive body image than women who underwent mastectomy for breast cancer.^55^–^59^ In addition, they were less likely to become self-conscious about body\break presentation or experience changes in body ideal and were more likely to retain perceptions of physical attractiveness and femininity.^55^,^58^ Women who underwent mastectomy alone felt less attractive and less sexually desirable and were less satisfied with their physical appearance.^58^,^60^ This is enhanced in younger women where body image may be more critical^35^,^61^ and mastectomy may, therefore, be more disruptive. On the other hand, some older women have been shown to care less about body image^62^ and find lumpectomy with radiotherapy more exhausting.^16^

Contrary to that, a study by Kraus^63^ showed a trend suggesting that women who had mastectomy were not less satisfied with their body image than women who had breast-conserving surgery. This outcome is supported by the finding that a woman's reaction toward mastectomy depends on her preoperation self-image.^64^ It is possible that patients who can cope with mastectomy differ psychologically compared with those who choose reconstruction, thus causing a selection bias. However, the small sample size of the cancer group does not allow generalization of the results,^65^ and a larger random sample drawn from the general population is required to confirm these results.

To show the negative effect of breast surgery on body image, Polivy^66^ conducted a prospective study in which he compared 3 types of surgical procedures: (1) breast biopsies for benign lesions, (2) mastectomies, and (3) general surgery. The results showed a significant decline in body image for women who had mastectomy at least 6 months after their surgery when compared with the other 2 procedures.

## SUMMARY

There are still many areas to improve in the quality of care given for breast cancer patients. Appropriate interventions should be implemented before and after the surgical procedure takes place. Conducting a randomized study that includes all treatment modalities is impossible nowadays, mainly because of ethical considerations. In the past, studies were carried out when patients were not aware of the equivalency of survival between mastectomy and breast-conserving surgery. Considering this, our efforts should be focused on the QOL of patients and on a proper psychological evaluation before and psychological support after the crisis. Behavioral techniques can be used to control the side effects of chemotherapy, like pain, nausea, and vomiting. Sexual adjustment could be addressed by an expert, along with complementary therapies, like massage or open discussions about the impact on sexuality.

It is in the hands of medical caregivers to look beyond the “cold” surgical treatment of breast cancer and implement a more holistic approach to patient care. Moreover, appropriate patient-reported questionnaires must be developed, specifically addressing cosmetic and reconstructive breast surgery. Only then new studies will obtain a meaningful basis on which surgical results can be compared.

## References

[1] Ries L, Harkins D, Krapcho M, Mariotto A, Miller B (2006). SEER Cancer Statistics Review 1975–2003.

[2] Worden JW, Weisman AD (1977). The fallacy in postmastectomy depression. Am J Med Sci.

[3] Fisher B, Bauer M, Margolese R (1985). Five-year results of a randomized clinical trial comparing total mastectomy and segmental mastectomy with or without radiation in the treatment of breast cancer. N Engl J Med.

[4] Jacobson JA, Danforth DN, Cowan KH (1995). Ten-year results of a comparison of conservation with mastectomy in the treatment of stage I and II breast cancer. N Engl J Med.

[5] Malata CM, Mcintosh SA, Purushotham AD (2000). Immediate breast reconstruction after mastectomy for cancer. Br J Surg.

[6] Goldwyn RM (1987). Breast reconstruction after mastectomy. N Engl J Med.

[7] Rowland JH, Holland JC, Chaglassian T, Kinne D (1993). Psychological response to breast reconstruction. Expectations for and impact on postmastectomy functioning. Psychosomatics.

[8] Donabedian A (1978). The quality of medical care. Science.

[9] Schipper H, Clinch J, Olweny C (1996). Quality of life studies: definitions and conceptual issues. Quality of Life and Pharmacoeconomics in Clinical Trials.

[10] Patrick D, Erickson P (1993). Health Status and Health Policy: Quality of Life in Health Care Evaluation and Resource Allocation.

[11] Franks PJ, Moffatt CJ (1998). Who suffers most from leg ulceration?. J Wound Care.

[12] Pusic AL, Chen CM, Cano S (2007). Measuring quality of life in cosmetic and reconstructive breast surgery: a systematic review of patient-reported outcomes instruments. Plast Reconstr Surg..

[13] Kerrigan CL, Collins ED, Striplin D (2001). The health burden of breast hypertrophy. Plast Reconstr Surg.

[14] Kerrigan CL, Collins ED, Kim HM (2002). Reduction mammaplasty: defining medical necessity. Med Decis Making.

[15] Mock V. (1993). Body image in women treated for breast cancer. Nurs Res.

[16] Pusic A, Thompson TA, Kerrigan CL (1999). Surgical options for the early-stage breast cancer: factors associated with patient choice and postoperative quality of life. Plast Reconstr Surg.

[17] Stevens LA, Mcgrath MH, Druss RG, Kister SJ, Gump FE, Forde KA (1984). The psychological impact of immediate breast reconstruction for women with early breast cancer. Plast Reconstr Surg.

[18] Hart S, Meyerowitz BE, Apolone G, Mosconi P, Liberati A (1997). Quality of life among mastectomy patients using external breast prostheses. Tumori.

[19] Rowland JH, Desmond KA, Meyerowitz BE, Belin TR, Wyatt GE, Ganz PA (2000). Role of breast reconstructive surgery in physical and emotional outcomes among breast cancer survivors. J Natl Cancer Inst.

[20] Wellisch DK, Dimatteo R, Silverstein M (1989). Psychosocial outcomes of breast cancer therapies: lumpectomy versus mastectomy. Psychosomatics.

[21] Moyer A (1997). Psychosocial outcomes of breast-conserving surgery versus mastectomy: a meta-analytic review. Health Psychol.

[22] Yurek D, Farrar W, Andersen BL (2000). Breast cancer surgery: comparing surgical groups and determining individual differences in postoperative sexuality and body change stress. J Consult Clin Psychol.

[23] Fallowfield LJ, Hall A (1991). Psychosocial and sexual impact of diagnosis and treatment of breast cancer. Br Med Bull.

[24] De Haes JC, Van Oostrom MA, Welvaart K. (1986). The effect of radical and conserving surgery on the quality of life of early breast cancer patients. Eur J Surg Oncol.

[25] Curran D, Van Dongen JP, Aaronson NK (1998). Quality of life of early-stage breast cancer patients treated with radical mastectomy or breast-conserving procedures: results of EORTC Trial 10801. The European Organization for Research and Treatment of Cancer (EORTC), Breast Cancer Co-operative Group (BCCG). Eur J Cancer.

[26] Nissen MJ, Swenson KK, Ritz LJ, Farrell JB, Sladek ML, Lally RM (2001). Quality of life after breast carcinoma surgery: a comparison of three surgical procedures. Cancer.

[27] Ganz PA, Rowland JH, Meyerowitz BE, Desmond KA (1998). Impact of different adjuvant therapy strategies on quality of life in breast cancer survivors. Recent Results Cancer Res.

[28] Van Dam FS, Schagen SB, Muller MJ (1998). Impairment of cognitive function in women receiving adjuvant treatment for high-risk breast cancer: high-dose versus standard-dose chemotherapy. J Natl Cancer Inst.

[29] Ganz PA, Desmond KA, Leedham B (2002). Quality of life in long-term, disease-free survivors of breast cancer: a follow-up study. J Natl Cancer Inst.

[30] Kiebert GM, Hanneke J, De Haes CJ (1990). Effect of peri-operative chemotherapy on the quality of life of patients with early breast cancer. Eur J Cancer.

[31] Palmer BV, Walsh GA, Mckinna JA, Greening WP (1980). Adjuvant chemotherapy for breast cancer: side effects and quality of life. Br Med J.

[32] Maguire GP, Tait A, Brooke M (1980). Psychiatric morbidity and physical toxicity associated with adjuvant chemotherapy after mastectomy. Br Med J.

[33] Schover LR, Yetman RJ, Tuason LJ (1995). Partial mastectomy and breast reconstruction. A comparison of their effects on psychosocial adjustment, body image, and sexuality. Cancer.

[34] Lindley C, Vasa S, Sawyer WT, Winer EP (1998). Quality of life and preferences for treatment following systemic adjuvant therapy for early-stage breast cancer. J Clin Oncol.

[35] Schover LR (1994). Sexuality and body image in younger women with breast cancer. J Natl Cancer Inst Monogr..

[36] Ganz PA, Rowland JH, Desmond K, Meyerowitz BE, Wyatt GE (1998). Life after breast cancer: understanding women's health-related quality of life and sexual functioning. J Clin Oncol.

[37] Meyerowitz BE, Watkins IK, Sparks FC (1983). Psychosocial implications of adjuvant chemotherapy. A two-year follow-up. Cancer.

[38] Arora NK, Gustafson DH, Hawkins RP (2001). Impact of surgery and chemotherapy on the quality of life of younger women with breast carcinoma: a prospective study. Cancer.

[39] Edsander-Nord A, Brandberg Y, Wickman M (2001). Quality of life, patients' satisfaction, and aesthetic outcome after pedicled or free TRAM flap breast surgery. Plast Reconstr Surg..

[40] Roth RS, Lowery JC, Davis J, Wilkins EG (2005). Quality of life and affective distress in women seeking immediate versus delayed breast reconstruction after mastectomy for breast cancer. Plast Reconstr Surg..

[41] Teimourian B, Adham MN (1982). Survey of patients' responses to breast reconstruction. Ann Plast Surg.

[42] Harcourt DM, Rumsey NJ, Ambler NR (2003). The psychological effect of mastectomy with or without breast reconstruction: a prospective, multicenter study. Plast Reconstr Surg.

[43] Dean C, Chetty U, Forrest AP (1983). Effects of immediate breast reconstruction on psychosocial morbidity after mastectomy. Lancet.

[44] Tarbox BB, Rockwood JK, Abernathy CM (1992). Are modified radical mastectomies done for T1 breast cancers because of surgeon's advice or patient's choice?. Am J Surg..

[45] Ayanian JZ, Guadagnoli E (1996). Variations in breast cancer treatment by patient and provider characteristics. Breast Cancer Res Treat.

[46] Wei JP, Sherry RM, Baisden BL, Peckel J, Lala G (1995). Prospective hospital-based survey of attitudes of Southern women toward surgical treatment of breast cancer. Ann Surg Oncol.

[47] Iscoe NA, Goel V, Wu K, Fehringer G, Holowaty EJ, Naylor CD (1994). Variation in breast cancer surgery in Ontario. CMAJ.

[48] Price B (1990). A model for body-image care. J Adv Nurs.

[49] Rytina S (2008). Imagined ugliness. Sci Am Mind.

[50] Salter M (1997). Altered Body Image: The Nurse's Role.

[51] Scott D, Eisendrath S (1986). Dynamics of the recovery process following initial diagnosis of breast cancer. J Psychosoc Oncol.

[52] Hopwood P (1993). The assessment of body image in cancer patients. Eur J Cancer.

[53] Ganz PA, Greendale GA, Petersen L, Kahn B, Bower JE (2003). Breast cancer in younger women: reproductive and late health effects of treatment. J Clin Oncol.

[54] Beckmann J, Johansen L, Richardt C, Blichert-Toft M (1983). Psychological reactions in younger women operated on for breast cancer. Amputation versus resection of the breast with special reference to body-image, sexual identity and sexual function. Dan Med Bull..

[55] Steinberg MD, Juliano MA, Wise L (1985). Psychological outcome of lumpectomy versus mastectomy in the treatment of breast cancer. Am J Psychiatry.

[56] Margolis GJ, Goodman RL, Rubin A, Pajac TF (1989). Psychological factors in the choice of treatment for breast cancer. Psychosomatics.

[57] Kiebert GM, De Haes JC, Van De Velde CJ (1991). The impact of breast-conserving treatment and mastectomy on the quality of life of early-stage breast cancer patients: a review. J Clin Oncol.

[58] Kemeny MM, Wellisch DK, Schain WS (1988). Psychosocial outcome in a randomized surgical trial for treatment of primary breast cancer. Cancer.

[59] Nano MT, Gill PG, Kollias J, Bochner MA, Malycha P, Winefield HR (2005). Psychological impact and cosmetic outcome of surgical breast cancer strategies. ANZ J Surg.

[60] Margolis G, Goodman RL, Rubin A (1990). Psychological effects of breast-conserving cancer treatment and mastectomy. Psychosomatics.

[61] Petrisek AC, Laliberte LL, Allen SM, Mor V (1997). The treatment decision-making process: age differences in a sample of women recently diagnosed with nonrecurrent, early-stage breast cancer. Gerontologist.

[62] Taylor ME, Perez CA, Halverson KJ (1995). Factors influencing cosmetic results after conservation therapy for breast cancer. Int J Radiat Oncol Biol Phys.

[63] Kraus PL (1999). Body image, decision making, and breast cancer treatment. Cancer Nurs..

[64] Goldberg P, Stolzman M, Goldberg HM (1984). Psychological considerations in breast reconstruction. Ann Plast Surg.

[65] Brewer M, Reis H, Judd C (2000). Research design and issues of validity. Handbook of Research Methods in Social and Personality Psychology.

[66] Polivy J (1977). Psychological effects of mastectomy on a woman's feminine self-concept. J Nerv Ment Dis.

